# FACTORS ASSOCIATED WITH ELECTRONIC HEALTH RECORD ADOPTION AMONG ADULT DAY SERVICES IN THE US

**DOI:** 10.1093/geroni/igad104.0103

**Published:** 2023-12-21

**Authors:** Yawen Li, Jay Chok, Geoffrey Chui, Don Roosan, Ken Shultz

**Affiliations:** California State University, San Bernardino, San Bernardino, California, United States; Keck Graduate Institute, Claremont, California, United States; California State University, San Bernardino, San Bernardino, California, United States; Western University of Health Sciences, Pomona, California, United States; California State University, San Bernardino, San Bernardino, California, United States

## Abstract

Successful adoption of Electronic Health Records (EHR) is becoming increasingly important for Adult Day Service Centers (ADSC) to function effectively and competently, particularly in care coordination with other healthcare providers. Therefore, in the present study, we assessed the EHR adoption among ADSC and its associated organizational characteristics using the 2018 National Study of Long-Term Care Providers (NPALS). The sample included 4,035 ADSC from a national representative sample. Directors or managers of ADSC completed the survey that included questions on organization characteristics, staffing, practice procedures, services, technology use, etc. Findings reveal that the adoption rate of EHR among ADSC was less than thirty percent and less than twenty percent of ADSC used EHR for health information exchange with either physicians or pharmacists. In addition, we found that an ADSC located near other healthcare providers, with Medicaid authorization and a higher percentage of Medicaid patients, and various computer capabilities such as recording participants’ demographic information, clinical notes, medications, service plans, and lab results were related to a higher likelihood of EHR use and EHR exchange. However, possibly due to privacy concerns, the capabilities to record participant problems and view lab results, negatively predict EHR exchange with physicians or pharmacists. The findings are discussed in terms of challenges and strategies to promote EHR adoption in ADSC.

